# The “New Synthesis”

**DOI:** 10.1073/pnas.2122147119

**Published:** 2022-07-18

**Authors:** Nicholas H. Barton

**Affiliations:** ^a^Institute of Science and Technology Austria, 3400 Klosterneuburg, Austria

**Keywords:** quantitative genetics, infinitesimal model, polygenic adaptation

## Abstract

When Mendel’s work was rediscovered in 1900, and extended to establish classical genetics, it was initially seen in opposition to Darwin’s theory of evolution by natural selection on continuous variation, as represented by the biometric research program that was the foundation of quantitative genetics. As Fisher, Haldane, and Wright established a century ago, Mendelian inheritance is exactly what is needed for natural selection to work efficiently. Yet, the synthesis remains unfinished. We do not understand why sexual reproduction and a fair meiosis predominate in eukaryotes, or how far these are responsible for their diversity and complexity. Moreover, although quantitative geneticists have long known that adaptive variation is highly polygenic, and that this is essential for efficient selection, this is only now becoming appreciated by molecular biologists—and we still do not have a good framework for understanding polygenic variation or diffuse function.

The rediscovery of Mendel’s work in 1900 led to the rapid development of classical genetics, and (after much turmoil) its integration with Darwin’s theory of evolution by natural selection, and with Galton’s statistical description of inheritance (1, 2). The past century has seen extraordinary and accelerating progress, yet key questions remain unresolved, for both evolutionary and molecular biology. In particular, it is not clear why Mendelian genetics predominates in eukaryotes, nor whether we will be able to attribute complex biological functions to specific genetic changes.

Following its publication in 1865 (3), Mendel’s discovery of the fundamental principles of inheritance remained unappreciated for 35 y—despite intense interest in inheritance, both for practical application and for its key role in evolution. The reasons for this are obscure (4), but one factor was that Mendel took an abstract and quantitative approach, which was quite alien to contemporary biology. Indeed, statistical models of biological systems have only recently begun to be developed in molecular biology (e.g., refs. 5 and 6).

The simultaneous rediscoveries of Mendel’s work in 1900 stimulated a burst of activity, which established classical genetics—showing that genes may carry multiple alleles, which interact to define phenotype, and that genes are linked on chromosomes, allowing them to be mapped in fine detail. This new field was seen as being in opposition to the biometric tradition initiated by Galton (7), and developed by Pearson and Weldon (2, 8, 9): Mendelian genetics dealt with mutations of large effect, whereas biometry described continuous variation. Thus, the early geneticists downplayed natural selection on slight variations [as had been emphasized by Darwin (10)], and saw mutation as the key evolutionary process.

Although this bitter dispute persisted into the midtwentieth century (1, 2), the resolution was known early on. Indeed, Mendel himself commented that continuous variation could be explained by a multitude of discrete factors, and similar comments were made by Bateson and Yule (2). The issue was resolved by experiments that showed artificial selection on continuous variation to be effective (2, 11), by careful dissection of apparently continuous traits (e.g., ref. 12) and, most important, by Fisher’s demonstration (13) that the correlations between relatives observed by the biometricians are consistent with Mendelian inheritance at multiple loci, and can be quantified by genetic components of variance.

During the 1920s, Fisher, Haldane, and Wright established the principles of population genetics, emphasizing that Mendelian genetics facilitates natural selection and, indeed, is an ideal foundation for it. Genes are replicated accurately, and passed on symmetrically through meiosis, so that variation is preserved, rather than being blended away (14, 15). This allows weak selection to be effective, given sufficient time. Adaptation is due solely to selection, which has a stronger influence on the course of evolution than mutation. Sexual reproduction brings together favorable alleles, and allows the efficient elimination of deleterious mutations. Thus, under the “New Synthesis” between the ideas of Mendel and Darwin, selection was seen as acting through the steady accumulation of slight favorable changes, with sex and recombination providing the immediate source of variation.

During the 1960s and 1970s, there were further conceptual developments—largely independent of the birth of molecular biology during the previous two decades (15). First, there was an understanding that adaptations cannot be explained simply as being “for the good of the species” (16, 17). One must explain how the genetic system (including sexual reproduction, recombination, and a fair meiosis, with each copy of a gene propagating with the same probability) is maintained through selection on individual genes, and remains stable despite mutations that would disrupt the system (17, 19, 20). Second, and related to this, there was an increased awareness of genetic conflicts that arise through sexual reproduction; selfish elements may spread through biased inheritance, even if they reduce individual fitness (19, 21, 22). In the decade following the discovery that DNA carries genetic information, all the fundamental principles of molecular biology were established: the flow of information from sequences of DNA through RNA to protein, the regulation of genes by binding to specific sequences in promoters, and the importance of allostery in allowing arbitrary regulatory networks (23, 24). Yet, the extraordinary achievements of molecular biology had little effect on the conceptual development of evolutionary biology. Conversely, although evolutionary arguments were crucial in the founding of molecular biology, they have had rather little influence in the half-century since (e.g., ref. 25). Of course, molecular biology has revealed an astonishing range of adaptations that demand explanation—for example, the diversity of biochemical pathways, that allow exploitation of almost any conceivable resource, or the efficiency of molecular machines such as the ribosome, which translates the genetic code. Technical advances have brought an accelerating flood of data, most recently, giving us complete genome sequences and expression patterns from any species. Yet, arguably, no fundamentally new principles have been established in molecular biology, and, in evolutionary biology, despite sophisticated theoretical advances and abundant data, we still grapple with the same questions as a century or more ago.

Although the foundations of evolutionary biology rest on the synthesis between Mendelian genetics and Darwinian evolution, and on the statistics of Galton and Pearson (2, 7, 8), these strands have remained quite separate. Population genetics has dealt largely with discrete Mendelian variation, and, in the 1960s, applied classical methods to study discrete molecular variation, revealed first in allozymes, and then DNA sequences. In contrast, biometry developed into quantitative genetics, which constructed a sophisticated statistical framework, largely driven by practical application to plant and animal breeding. The connection with evolutionary biology was restored in the 1960s and 1970s, through work by Robertson (26), Hill and Robertson (27), Bulmer (28), and Lande (29, 30), but quantitative genetics remains a distinct subfield. Indeed, even from a theoretical point of view, much remains to be done to connect the statistical methodology of quantitative genetics with its foundation in Mendelian population genetics (31).

Bearing this historical overview in mind, we now turn to two questions that remain unresolved, a century after the New Synthesis: Why Mendelian genetics? How can we understand adaptation that is based on very many interacting genes?

## Why Mendelian Genetics?

Almost all eukaryotes reproduce sexually, through a meiosis which generates haploid gametes from a diploid cell. Although ubiquitous, the process has obvious costs: the biochemical cost of meiosis itself, the difficulty in finding a mate, the indirect costs that come from shuffling genes that had been selected to work well, the danger from horizontal spread of selfish elements, the consequences of sexual selection, and so on (17). Most fundamentally, if (as is usually the case) females provide most of the resources of offspring, they could pass on twice as many copies of their genes by only producing identical daughters, rather than helping propagate the genes of their sexual partner. This is termed the “twofold cost” of sex (17). There is a consensus that the most likely explanation for the prevalence of sexual reproduction is that it facilitates efficient natural selection. This idea was suggested by Weissman (32), and widely accepted. However, in the 1970s, Maynard Smith (17), Williams (18) and others realized that, even if sexual reproduction is essential for a species to adapt, this does not explain why it is maintained at a high level: Why do asexual individuals not displace sex, as indeed may happen in the short term? Why are rates of recombination kept high, even though we know that selection could change them?

It took a good deal of theoretical effort to show that sex and recombination can gain a substantial advantage at the level of individuals by breaking up negative associations between selected alleles—that is, by bringing together favorable alleles that tend to be on different genomes (33). Although this explanation is compelling from a theoretical point of view, it remains unclear whether there is, in fact, enough selection acting for it to apply. More precisely, there must be sufficient heritable variance in fitness that selection causes a substantial increase in mean fitness in every generation—an increase that compensates for mutation and for an environment that is always deteriorating, as conditions (biological and physical) change. Several elegant manipulative experiments suggest that sex does indeed have a short-term advantage, but it is quite unclear what is the magnitude or source of the heritable fitness variance that is responsible for this advantage (34, 35).

A closely related, and quite fundamental, puzzle has received less attention: Why is meiosis almost always fair, with each copy having the same chance to propagate, so that neither can gain an advantage by exploiting meiosis? There are many examples where “selfish genes” break this symmetry, and, in the short term, they may be successful: through biasing meiosis itself (meiotic drive), killing homologous genes (segregation distortion), or transposing across the genome—which gives a long-term advantage only if sexual reproduction then allows spread out of individual lineages. Correspondingly, an extraordinary fraction of molecular mechanism seems to be devoted to countering such elements, and their side effect may impose a serious load (19, 21). It may be that the flexibility of the nuclear genome—the “parliament of genes” (36)—usually allows the suppression of selfish elements, but this is hard to establish.

Unicellular life evolved long before the invention of meiosis by eukaryotes, and bacteria and Archaea thrive without it. Thus, although sexual reproduction together with fair rules for genetic segregation seem to be essential for the long-term survival of eukaryote species, they are not universally required. A plausible explanation is that bacteria and Archaea have very large population sizes, short generation times, and a variety of irregular mechanisms for recombination. In population genetics, the effective rate of recombination is determined by the product of the effective population size and recombination rate, N_e_r, which may be as high in “asexuals” as in organisms with regular sex (37). Although bacteria and Archaea have not evolved complex development or multicellularity, they share astonishingly precise molecular machinery, and a sophisticated biochemical repertoire. The numbers of genes in any one individual are lower than in typical eukaryotes, but the ranges overlap, and the number of genes available in the wider population [the “pan-genome”; (38)] may be much larger. Nevertheless, it seems that eukaryotes require sexual reproduction in order to maintain their complexity, despite relatively small population size and long generation times. It is remarkable that complex eukaryotes can hold their own against their far more numerous and fast-replicating competitors.

## How Can We Understand Polygenic Adaptation?

Theoretical arguments for the ubiquity of polygenic adaptation were supported by the observation that artificial selection, on almost any trait and almost any population, causes a steady response, which continues for at least hundreds of generations if the population is sufficiently large (11, 39). Direct observation of the variance generated by new mutations shows that the response is largely due to initial variation, which is reshuffled by recombination to give novel genotypes not present in the original population. Over the past decade, this basic principle has been confirmed by large-scale genome-wide association studies, which show that, although trait variation can be allocated to individual single-nucleotide polymorphisms (SNPs), there are typically enormous numbers of them, and even the largest surveys can hardly allocate the bulk of the variance to specific SNPs (40). Boyle et al. (41) propose an “omnigenic” model: Although some fraction of variance is due to “core” genes, in pathways directly responsible for the trait, the bulk of genetic variance is due to effects that are spread over the whole genome—or, at least, over all parts of the genome that are expressed in the relevant tissue and life stage.

These observations are entirely consistent with the “infinitesimal model,” which is the foundation of quantitative genetics (42). The origin of this term is obscure: It is associated with Fisher (13), but the term “infinitesimal model” came to be used only later (e.g., ref. 43). In fact, Galton (7) stated the essence of the infinitesimal model: He noted that the distribution among offspring is independent of the trait values of their parents, and used this to calculate the equilibrium variance under stabilizing selection—a calculation next published a century later (43).

At the phenotypic level, the infinitesimal model states that the heritable variance among siblings is normally distributed with a variance that is independent of the parents’ traits, and can be calculated from the relatedness between uniting gametes. Fisher (13) justified this assumption by showing that it holds when traits are the sum of effects of very many loci. However, it applies much more generally, even with interaction between genes [i.e., dominance and epistasis (42)]. The key assumption is that variation within the population is small, relative to the extremes of what can evolve from existing standing variation, so that knowing the trait value of an individual gives little information about the genotype of its offspring.

The infinitesimal model is the foundation for the “animal model,” which predicts an individual’s phenotype, based on the phenotypes of its relatives; this is the basis for efficient breeding programs. The advent of DNA-based genetic markers in the 1990s, followed by whole-genome sequencing, raised hopes that quantitative trait loci (QTL) could be mapped and then manipulated to improve yields. However, it soon became apparent that, for most traits, there are far too many QTL for this to be feasible. Instead, genetic markers are used to improve the accuracy of predictions, thereby increasing the rate of improvement—but without identifying specific loci. This approach, known as “genomic selection” (44), has had the greatest impact in dairy cattle, doubling the rate of increase in mean milk yield (39). This success is partly due to the ability to predict a bull’s breeding value from his female relatives, but there is a substantial gain even where an individual’s phenotype can be directly measured.

## Implications of the Infinitesimal Model

What does the success of the infinitesimal model imply about the alleles responsible for that variation? Robertson (26) showed that the total change in a trait caused by selection is limited by effective population size, equaling 2*N_e_* times the change in the first generation. This is simply because, under the infinitesimal model, the genetic variance declines solely due to inbreeding, and so persists for ∼2*N_e_* generations. This simple argument applies even with dominance and epistasis (45), and fits observations remarkably well (46). Robertson (26) showed that this limit can be understood as a consequence of a slight bias in the probability of fixation of the underlying alleles, since the ultimate change in mean is just the sum of the effect of each allele, times the difference between its fixation probability and its initial frequency. Crucially, this argument implies that, under the infinitesimal model, genetic variation is based on alleles for which drift dominates selection (i.e., the product of effective population size and selection coefficient, *N_e_s*, is less than one). The infinitesimal model can be understood as a slight bias away from neutrality, due to selection that is spread over very many loci.

When understood in this way, the infinitesimal model has radical implications. First, the “signature of selection” at individual loci will be very weak, making it impossible, in principle, to identify, from sequence data, the loci responsible for adaptation. Second, while the genome sequence can increase the accuracy of the predicted phenotype, as in genomic selection, there is a fundamental limit to the accuracy that is possible (47, 48). Finally, it raises the possibility that biological function is itself diffuse, and not necessarily mediated by identifiable pathways.

Of course, we have many striking examples where trait differences are largely due to a few major loci, and we have examples where selective sweeps or balancing selections have been identified and validated from sequence data. However, these tend to be in particular kinds of trait (for example, pesticide resistance or pigmentation) which we expect to have a simple Mendelian basis (49). It is not clear what fraction of adaptation is attributable to such “simple” loci, or what fraction of selection can be detected through analysis of DNA sequence at specific loci.

Even though the infinitesimal model describes the evolution of polygenic traits over hundreds of generations, it is unlikely to apply over evolutionary timescales. In a fully infinitesimal world, the genetic covariance, **G**, would reach a neutral equilibrium between mutation and drift, 2*N_e_V_m_*, where ***V_m_*** is the mutational covariance introduced by mutation in each generation. While it may well be that the bulk of sequence variation is effectively neutral, with diversity shaped by the combined effect of mutation and drift (taking into account the effects of population structure and linked selection), this seems implausible for selected traits—although not altogether easy to refute.

An obvious argument is that genetic variance should be proportional to effective population size, which can hardly be the case. However, just the same argument applies to sequence variation, and the resolution may be that selective sweeps set an upper limit to the lifetime of variation (50). A more robust prediction, then, is that additive genetic variance should be proportional to sequence diversity, in comparisons across populations and species. Conversely, trait variance should not depend on the strength of stabilizing selection on the trait. It is not clear that this argument rules out a neutral theory for trait variance.

One might, however, consider a modification of the infinitesimal model, which supposes that genetic variance is not influenced by selection on the trait of interest. One imagines that sequence variation includes alleles that are deleterious either intrinsically or because they perturb traits under stabilizing selection. Such alleles will tend to be rarer than expected if there were no selection, and they may experience substantial *N_e_s* overall. However, we might still suppose that they contribute to genetic variance in the trait of interest, and that heritable variance in the focal trait might be broadly proportional to sequence diversity, independent of their effect on the focal trait. Studies of the standing genetic covariance, **G**, and the mutational covariance, ***V_m_***, for high-dimensional traits such as gene expression and wing shape in *Drosophila* suggest widespread pleiotropy and constraint (40, 51), but we are far from understanding whether and how heritable variation may be maintained by a balance between mutation and stabilizing selection.

**Fig. 1.**
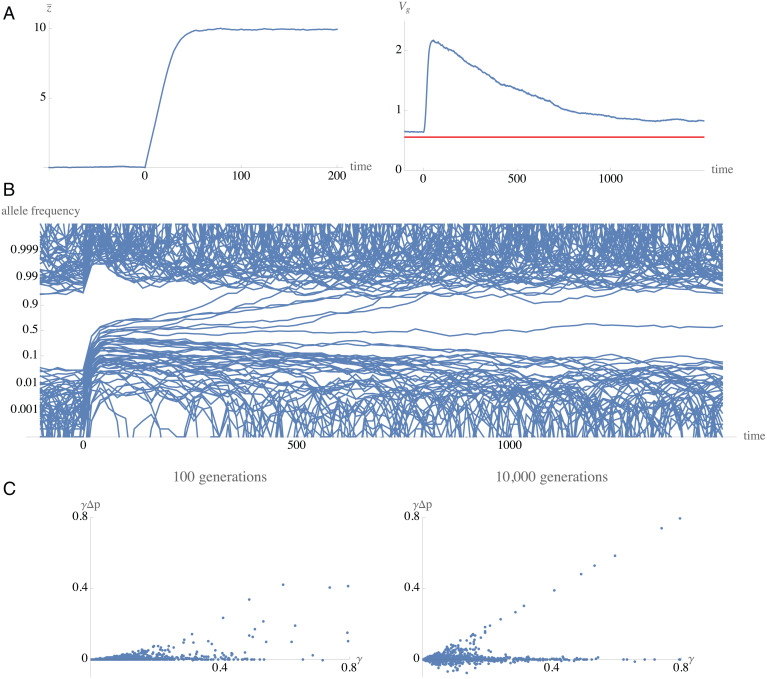
Response of a trait, and of the underlying allele frequencies, to a change in optimum from *z*_opt_ = 0 to 10 at time zero. (*A*) Trait mean (*Left*) and variance over time (*Right*). The mean responds rapidly, reaching the new optimum within 50 generations. The variance increases sharply, as + alleles increase in frequency, and then gradually returns to its original value over thousands of generations, as stabilizing selection acts to reduce the variance. (*B*) Frequencies of the + allele, plotted for the 95 loci with effect γ>0.3; the plot is on a logit scale, so as to expand frequencies near to fixation. Allele frequencies increase sharply as the mean moves to the new optimum, but then slowly return toward fixation, as stabilizing selection acts to reduce heterozygosity. Ultimately, only seven of these alleles with γ>0.2 substitute, contributing a change in mean of 3.82 out of the total of 10. (*C*) The contribution of each locus to the change in trait mean, γΔp, plotted against its effect. By 100 generations after the change in optimum (*Left*), only the largest-effect alleles have shifted substantially; alleles with effect γ>0.2 account for a change in mean of 4.1 out of a total 9.6, with seven loci contributing 2.0. Ultimately (*Right*), large-effect loci have either fixed (upper diagonal line, Δp = 1) or returned to low frequency (Δp ≈ 0); somewhat less than half the ultimate change in mean (3.8 out of ∼10) is contributed by substitutions of large-effect alleles, the remainder being due to small shifts in frequency of weakly selected alleles. There are 1,000 biallelic loci, with additive effects γ drawn from an exponential distribution with mean 0.12. Mutation is symmetric, at rate μ = 0.000025; fitness is $∼\text{exp}[{\left(z-z_{\text{opt}}\right)}^{2}/2V_{s}].$, with *V_s_* = 20. There are *n* = 10^4^ haploid individuals. The simulation is run for 10^4^ generations, to reach equilibrium, when the mean is close to the optimum, and the genetic variance maintained in a balance between mutation, drift, and stabilizing selection is *V_g_* = 0.556; this is close to the prediction from the diffusion approximation [red line in *A*, *Right* (ref. 53, equations 6 and 7)]. See *SI Appendix* for details.

Even if organisms do not evolve in a fully infinitesimal world, weakly selected alleles may be required for an efficient response to changing conditions, and the consequent changes in allele frequency may be inscrutable even if *N_e_s* ≫ 1. The genetic response to a change in optimum under stabilizing selection has been studied by Höllinger et al. (52), and by Hayward and Sella (53); Fig. 1 shows a simulated example of the response to a large shift in mean, by ∼12 genetic SDs. Initially, the change in mean is proportional to the genetic variance. Under a balance between mutation and stabilizing selection, the contribution of alleles to genetic variance is independent of their effect on the trait (above a small threshold), and, therefore, we expect an initial contribution from weakly selected alleles. However, large-effect alleles quickly rise in frequency, and start to contribute more of the genetic variance, and, therefore, more of the change in mean. The mean quickly approaches the new optimum (Fig. 1*A*), but, as it does so, selection on the underlying alleles shifts from directional to stabilizing. More precisely, when the deviation from the optimum becomes smaller than the allele’s effect, selection acts primarily to reduce genetic variance, and, hence, against heterozygotes. Therefore, the larger-effect alleles decrease back to low frequency, unless they have become common enough to go on to fixation (Fig. 1*B*). There follows a long period during which allele frequencies return to near-fixation, and the genetic variance returns to its original equilibrium; the mean will have changed through substitution of a few alleles, but also through frequency changes at very many more loci, in a highly unpredictable way. Thus, even in this very simple model, a smooth and predictable change in the trait mean is mediated by complex change at a myriad of underlying loci. This is far from the infinitesimal regime—the genetic variance does change as a result of selection—but it would not be easy to infer the genetic basis of trait change, even with complete knowledge of allele frequencies.

If we consider a single allele, then we can see it as “effectively neutral” if its effect on fitness is less than ∼1/2*N_e_*. This idea was used by Ohta (54) in a modification of the neutral theory, to suggest why larger populations might be less diverse than expected (because a smaller fraction of mutations would be effectively neutral), and why rates of substitution might be constant per year rather than per generation (because species with shorter generation times might tend to have large populations, and have a smaller fraction of effectively neutral mutations that contribute to long-term evolution). Lynch (21) has applied this concept to argue that molecular adaptations that are under weak selection cannot be established or maintained in (relatively) smaller populations, imposing a “drift barrier” to adaptation. Along the same lines, Kondrashov (55) has argued that deleterious mutations with *N_e_s* ≈ 1 will accumulate, steadily degrading the population. Both ideas seem problematic if we view adaptation as due to optimization of polygenic traits: Organisms can be well adapted even if drift dominates selection on individual alleles, and, under a model of stabilizing selection on very many traits, any change that degrades fitness can be compensated.

## The Efficiency of Adaptation

Arguably, and perhaps counterintuitively, adaptation is most efficient in an infinitesimal world. The extent of selection is limited by the reproductive capacity of the organism, which we can measure by the variance in absolute fitness (i.e., number of offspring). The mean absolute fitness must be two for a stable sexual population, and the variance is bounded by the maximum possible number of offspring. Imagine directional selection on an additive trait. What “genetic architecture” will maximize the number of favorable substitutions? Strong selection on each of a few loci will give a rapid response, but limited to those few loci. As we increase the number of selected loci, *N_e_s* becomes smaller, and fixation is no longer certain. Nevertheless, the overall expected number of favorable substitutions is maximized in the infinitesimal limit, when fixation probability is only slightly biased toward the favored alleles. Systematically negative epistasis (for example, truncation selection) can increase efficiency, but arbitrary epistasis will randomize the marginal effect of each allele, and so make selection less efficient (56).

This argument can be generalized, and made more precise, by measuring progress by the information gained (57). We imagine that there is some relatively small set of fit genotypes, and ask how effectively selection can compress the distribution of genotypes onto that set. The degree of compression is measured by the Kullback–Liebler divergence between the actual distribution, and, in the absence of selection, this measure can be applied to the distribution of individual phenotypes, individual genotypes, or genotype frequencies across a population; it is the latter that is appropriate here. Remarkably, the gain in information is bounded by the product of the effective population size and the cost of selection—the latter being defined as an information measure that is itself bounded by the maximum reproductive capacity, or the variance in fitness (58). This is a much looser constraint than that set by the substitution or lag load, which does not involve the effective population size (57). The information gain is limited by drift, but is achieved when individual alleles are nearly neutral. Note also that the rate of progress, as measured by the rate of gain of information, is proportional to *N_e_* in a sexual population, but only to log(*N_e_*) in asexuals: Free recombination is needed to allow selection to exploit the independent information that comes from the birth or death of each individual.

There are several distinct reasons why adaptation is most efficient when based on slight variations. As Darwin so clearly argued, complex adaptation is most likely to evolve through the accumulation of slight variations (10): If mutation is random with respect to adaptation, then variants of large effect are likely to disrupt function. This argument was quantified by Fisher’s geometric model, under which the typical size of favorable mutations scales inversely with the square root of the number of available dimensions (59, 60). A second argument is that weakly selected variants allow populations to find the best combination of alleles, without being trapped at suboptimal “adaptive peaks.” Finally, and most generally, selection on slight variations accumulates information most efficiently, given a limited reproductive capacity.

## Open Questions

### What Kind of Selection Is Responsible for Adaptation?

One of the most remarkable (yet unappreciated) biological phenomena is the ubiquity of genetic variation. The high heritability of quantitative traits has long been known, while the corresponding extent of molecular variation was discovered relatively recently (36). It is now clear that the traits that matter to us, and to the organism, are influenced by an extremely large number of genetic loci (41). This implies that the immediate response to selection is also highly polygenic. However, we still do not have a good quantitative understanding of the nature of variation. What is the distribution of selection coefficients that drive adaptation? How extensive are pleiotropy and epistasis? To what extent is adaptation based on new mutations, that arise during the response to selection, rather than on mutations that had been segregating for a long time, as part of the standing variation?

What is the relation between the immediate response to selection, which is due to changes in allele frequency at very many loci, and the longer-term response, manifest in fixed differences between species? The infinitesimal model does not require that long-term divergence is based on subtle shifts in allele frequencies, although that would be possible in principle. Traits might diverge by many SDs, without any fixed differences at all, and sister species may share variation across most of their genome [e.g., as do *Drosophila persimilis* and *Drosophila pseudoobscura* (61)]. However, with enough divergence, there can hardly be enough shared polymorphism for this to be possible. Rather, the infinitesimal model implies that substitutions are slightly biased toward favorable changes (26); traits may, as a result, be quite distinct, but that divergence could have been achieved by many alternative sets of allelic substitutions.

Genetic incompatibilities between species are often strongly selected. Detailed study of incompatibilities between *Drosophila* species, using the methods of classical genetics, have mapped these strong incompatibilities in great detail, showing how interactions between a few loci cause severe infertility or inviability (62). However, divergence may nevertheless have been driven by weak selection, or even have been entirely neutral. Bateson, Dobzhansky, and Muller independently proposed a simple genetic model, in which derived alleles cause strong incompatibilities when they come together in hybrids (63); yet, these incompatibilities need never be expressed during divergence. Hybrids have genotypes that have never been tested by selection, and so are expected to be less fit than the small fraction of genotypes that link the divergent species via their common ancestor, which were necessarily fit. A simple model is that any pair of alleles has some small probability of causing a strong incompatibility, so that incompatibility accumulates at least quadratically with divergence (63). This probability is estimated to be extremely small; it is remarkable that hybrids between organisms that differ at thousands of sites are usually compatible, so that hybrids remain fit even after millions of years of divergence, reflecting the robustness of organisms to genetic perturbation. Extending this argument, finding that most mutations degrade a gene’s function, often substantially, does not imply that the function itself was built up by strong mutations. In quantitative terms, the distribution of effects on fitness of random mutations is not the same as that of adaptive substitutions.

### Can We Predict Phenotype from Genotype?

The highly polygenic basis of trait variation makes it practically impossible to reliably attribute it to individual variants. Nevertheless, we can make statistical predictions of phenotype from the genotype as a whole, by summing estimated individual effects into a “polygenic score.” This approach is the basis for “genomic selection,” which has substantially improved the effectiveness of artificial selection, by improving the accuracy of estimates of breeding value (44). However, this approach requires very large samples to estimate effects of genomic segments, and these estimates apply only to that population. In humans, attempts to infer selection on traits from changes in polygenic score through time, or between populations, have been confounded by cryptic population structure (40, 64, 65). Even setting aside these difficulties, it is not clear how useful statistical predictions can be in other contexts. In medicine, they may improve the efficiency of screening programs, where costs and benefits must be balanced. However, for an individual patient, improving the statistical estimates of the risk of multiple diseases will rarely be helpful. Similarly, in functional studies, it had been hoped that effects might at least be concentrated in certain functional pathways; yet, this seems not to be generally true. Under the omnigenic model (41), the aggregate effects of distantly connected genes are responsible for more variance than the smaller number of interactions with genes that have a close functional connection.

### What (if Anything) Does the Prevalence of Polygenic Variation Imply for Our Understanding of Function?

The simplest view of how organisms function would involve a minimal number of strong interactions, with subfunctions performed by distinct modules; we might imagine that this would be the kind of organism produced by an intelligent designer. Yet, function might also be diffuse, being spread over many very weak interactions, with overlapping subfunctions. The most extreme example that comes to mind could be where the total DNA content regulates cell size, such that the function is spread evenly over the whole genome. Less extreme would be where gene expression is regulated by the weak binding of transcription factors at many sites; such diffuse regulation seems widespread in eukaryotes (e.g., ref. 66).

The polygenic basis of genetic variation and of adaptation does not necessarily imply that function itself is diffuse: Selection on slight variants might have gradually perfected tight binding at a single site. Nevertheless, the analogy is suggestive, and the converse implication should hold: If function were typically diffuse, then selection on each component would be weak, and pleiotropy would be widespread.

There has been much discussion of the evolution of evolvability and of robustness; one expects that diffuse function could more easily evolve, and might be more robust to disruption (67, 68). However, it would be hard to demonstrate that diffuse function evolved for these reasons—just as it has been hard to demonstrate that sexual reproduction evolved as an adaptation to facilitate natural selection (17). My argument here is that, if adaptation is largely infinitesimal, that suggests (but does not require) that function may also be diffuse. There is a strong bias toward elucidating the simplest systems (i.e., adaptations based on a few major alleles, and mechanisms based on sparse functional interactions), making it hard to judge what is typical.

## Conclusions

Mendelian genetics—with fair segregation and regular recombination at meiosis—may have been crucial in allowing complex eukaryotes to evolve. When Mendel’s work was rediscovered in 1900, the emphasis was on genetic variants with major phenotypic effects; to a large extent, the same approach still dominates. Yet, Mendelian genetics facilitates natural selection most effectively when it acts through a miasma of slight variants. Paradoxically, therefore, natural selection may be most effective in cases where it is least accessible to investigation. If adaptation is polygenic, and function is diffuse, we require a quantitative approach such as that pioneered by Mendel, even if it cannot attempt to elucidate the effects of each distinct Mendelian variant.

## Supplementary Material

Supplementary File

## Data Availability

All study data are included in the article and/or supporting information.
