# Supplementary material for: The “New Synthesis”
Source: Proc Natl Acad Sci U S A. 2022 Jul 18;119(30):e2122147119. doi: 10.1073/pnas.2122147119 (PMC9335343; doi:10.1073/pnas.2122147119)
Supplement: Supplementary File [file pnas.2122147119.sapp.pdf]

Example: $N = 10^4$, $n = 10^3$, $\alpha \sim \frac{4}{\sqrt{n}}$, $V_s = 20$, $\mu = 2.5 \times 10^{-5}$, $z_{opt} = 0 \to 10$, $T = 10^4 + 10^4$

Setting up

Choose $N = 10^4$ individuals, $n = 10^3$ loci, and γ from an exponential, mean $\frac{4}{\sqrt{n}}$. The range is ±60.8, and the maximum possible genetic variance would be 7.34. I run this for 10^4 generations with $V_s = 20$, $\mu = 2.5 \times 10^{-5}$, optimum 0, and then switch to optimum 5 for 10^4 generations.

```
ni = 10^4; nl = 10^3; mu = 2.5 × 10^-5;
θ0 = 0; θ2 = 10; Vs = 20; ϵ = 10 / ni; t1 = 10^4; t2 = 10^4;
a = RandomReal[ExponentialDistribution[√nl / 4], nl];
{Total[Abs[a]] / 2, Total[a^2] / 4, MinMax[a]}
```

```
Out[∘]= {60.7838, 7.66107, {0.000588235, 0.796693}}
```

This sets up the first 10^4 generations at optimum 0, and then another 10^4 generations at optimum 10:

```
time = 0;
p0 = initP[2 ni mu, ni/Vs, a, ni];
Timing[pl = NestList[(time += 1;
      iterate[#, θ0, a, Vs, mu, ni, ϵ]) &, p0, t1];
pl2 = Join[Drop[pl, -1], NestList[(time += 1;
       iterate[#, θ2, a, Vs, mu, ni, ϵ]) &, pl⟦-1⟧, t2]];]
```

```
Out[∘]= {89.7929, Null}
```

This saves the allele counts (pl2), the allelic effects (a) and the initial counts (p0):

```
In[∘]:= Save["pl2, a, p0 28.3.22", {pl2, a, p0}];
```

Example figure

This is the predicted equilibrium variance, from the diffusion approximation. This is substantially less than the deterministic House of Cards approximation, $2 V_s \sum \mu$

```
In[∘]:= vpred = Total[(#^2/4 exp4pq[ni mu / 2, ni #^2/(2 Vs)]) & /@ a];
        {vpred, 2 nl mu Vs}
```

```
Out[∘]= {0.558941, 1.}
```

The top row shows the change in mean after 100 generations and after 10^4 generations, then V_g initially, after 100 generations, and ultimately. The next row shows the contributions of the 288 alleles with effect γ>0.15. The last row shows the contributions of the 95 large effect alleles (γ>0.3).

In[∘]:=
```
xx = Pick[Range[nl], (# > 0.3) & /@ a];
xxs = Pick[Range[nl], (# > 0.15) & /@ a];
pq0 = pl2[[t1]]/ni (1 - pl2[[t1]]/ni);
pq100 = pl2[[t1 + 100]]/ni (1 - pl2[[t1 + 100]]/ni);
pq∞ = pl2[[-1]]/ni (1 - pl2[[-1]]/ni);
Prepend[{Length[#], a[[#]].dp100[[#]], a[[#]].dp[[#]], a[[#]]^2.pq0[[#]],
      a[[#]]^2.pq100[[#]], a[[#]]^2.pq∞[[#]]} & /@ {Range[nl], xxs, xx} // N,
   {"# loci", "Δz̅ at 100", "Δz̅ at ∞", "Vg init", "Vg at 100", "Vg at ∞"}] //
 TableForm
```

Out[∘]//TableForm=

# loci	$\overline{\Delta z}$ at 100	$\overline{\Delta z}$ at ∞	V_g init	V_g at 100	V_g at ∞
1000.	9.61555	9.68176	0.643834	2.09163	0.636374
288.	6.73501	6.24588	0.309973	1.76478	0.32562
95.	4.11229	3.8463	0.0803577	1.35302	0.104925

The mean adjusts very rapidly, but the variance equilibrates very slowly. The diffusion approximation to the variance (red) fits very well, and is substantially lower than the deterministic HoC prediction of $2\, V_s \sum \mu_i = 1$. There is a transient doubling of variance, which decays much more slowly than the change in mean. Note that the lower right panel only shows the decay of V_g over 1500 generations, during which time substitutions are complete - but it decays further over the subsequent 5000 generations.

In[∘]:=
```
bs = {FontFamily → "Times", FontSize → 14};
tt = Round[0.99 t1] ;; Round[1.15 t1] ;; 20;
GraphicsRow[{ListLinePlot[
   Transpose[{Range[-100, 200], zbar[#, a, ni] & /@ pl2⟦(t1 - 100) ;; (t1 + 200)⟧}],
   BaseStyle → bs, Ticks → {{0, 100, 200}, {0, 5, 10}},
   AxesOrigin → {-100, 0}, AxesLabel → {"time", "z̄"}, AspectRatio → 0.5],
  Show[ListLinePlot[Transpose[{Range[-100, 1500],
      Vg[#, a, ni] & /@ pl2⟦(t1 - 100) ;; (t1 + 1500)⟧}],
    PlotRange → {{-100, 1500}, {0, 2.5}}],
   Plot[vpred, {x, -100, 1500}, PlotStyle → Red], AspectRatio → 0.5,
   Ticks → {{0, 500, 1000}, {0, 1, 2}},
   AxesOrigin → {-100, 0}, AxesLabel → {"time", "Vg"}, BaseStyle → bs]}]
```

Out[∘]=

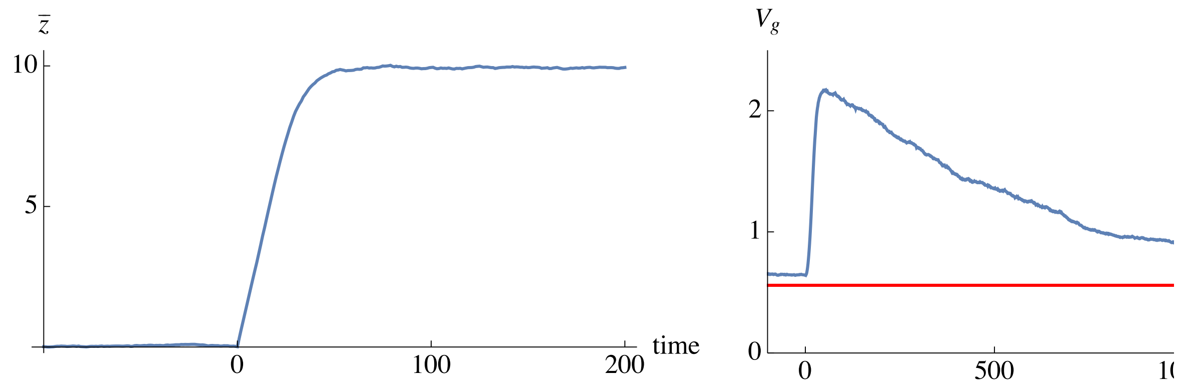

In[•]:=
```
Show[ListLogPlot[Transpose[{Range[-t1, t2]⟦tt⟧, (0.01 + pl2⟦tt, #⟧)/(0.01 + ni - pl2⟦tt, #⟧)}],
    PlotRange → {All, {0.0001, 10 000}}, Joined → True, AxesOrigin → {-100, 0.0001},
    Ticks → {{0, 500, 1000}, {{1/999, "0.001"}, {1/99, "0.01"}, {1/9, "0.1"},
      {1, "0.5"}, {9, "0.9"}, {99, "0.99"}, {999, "0.999"}}}] & /@ xx,
  AspectRatio → 0.3, BaseStyle → bs, AxesLabel → {"time", "allele frequency"}]
```

Out[•]=

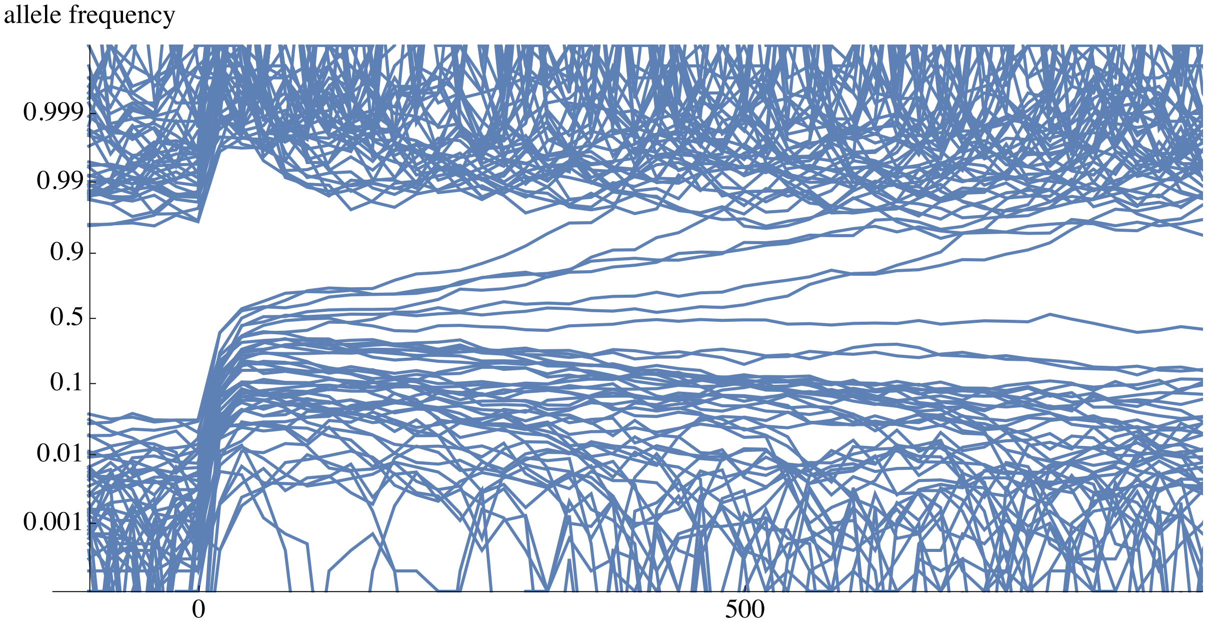

In[•]:=
```
GraphicsRow[
  ListPlot[Transpose[{a, #⟦1⟧}], PlotStyle → PointSize[0.01], AxesLabel →
      {"γ", "γΔp"}, PlotLabel → #⟦2⟧, PlotRange → {{0, 0.8}, {-0.1, 0.8}},
    BaseStyle → bs, Ticks → {{0, 0.4, 0.8}, {0, 0.4, 0.8}}] & /@
   {{a dp100, "100 generations"}, {a dp, "10,000 generations"}}]
```

Out[•]=

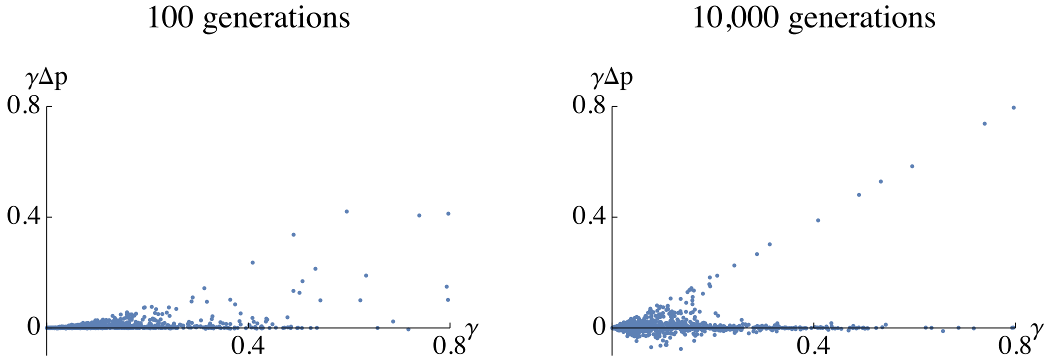

The figure itself

Figure 1. Response of a trait, and of the underlying allele frequencies, to a change in optimum from z_{opt} = 0 to 10 at time zero. a) Trait mean and variance over time (left, right). The mean responds rapidly, reaching the new optimum within 50 generations. The variance increases sharply, as + alleles increase in frequency, and then gradually returns to its original value over thousands of

generations, as stabilising selection acts to reduce the variance. b) Frequencies of the + allele, plotted for the 95 loci with effect γ>0.3; the plot is on a logit scale, so as to expand frequencies near to fixation. Allele frequencies increase sharply as the mean moves to the new optimum, but then slowly return towards fixation, as stabilising selection acts to reduce heterozygosity. Ultimately, only 7 of these alleles with γ>0.2 substitute, contributing a change in mean of 3.82 out of the total of 10. c) The contribution of each locus to the change in trait mean, $\gamma\Delta$p, plotted against its effect. By 100 generations after the change in optimum (left), only the largest effect alleles have shifted substantially; alleles with effect γ>0.2 account for a change in mean of 4.1, out of a total 9.6, with 7 loci contributing 2.0. Ultimately (right plot), large-effect loci have either fixed (upper diagonal line, Δp=1) or returned to low frequency (Δp~0); somewhat less than half the ultimate change in mean (3.8 out of ~10) is contributed by substitutions of large-effect alleles, the remainder being due to small shifts in frequency of weakly selected alleles. There are 1000 biallelic loci, with additive effects γ drawn from an exponential distribution with mean 0.12. Mutation is symmetric, at rate $\mu = 2.5 \times 10^{-5}$; fitness is $\sim \exp\left[-(z - z_{\text{opt}})^2 / (2\, V_s)\right]$ with $V_s = 20$. There are $N = 10^4$ haploid individuals. The simulation is run for 10^4 generations, to reach equilibrium, when the mean is close to the optimum, and the genetic variance maintained in a balance between mutation, drift, and stabilising selection is $V_g = 0.556$; this is close to the prediction from the diffusion approximation (red line at top right; 23, Eqs. 6, 7).

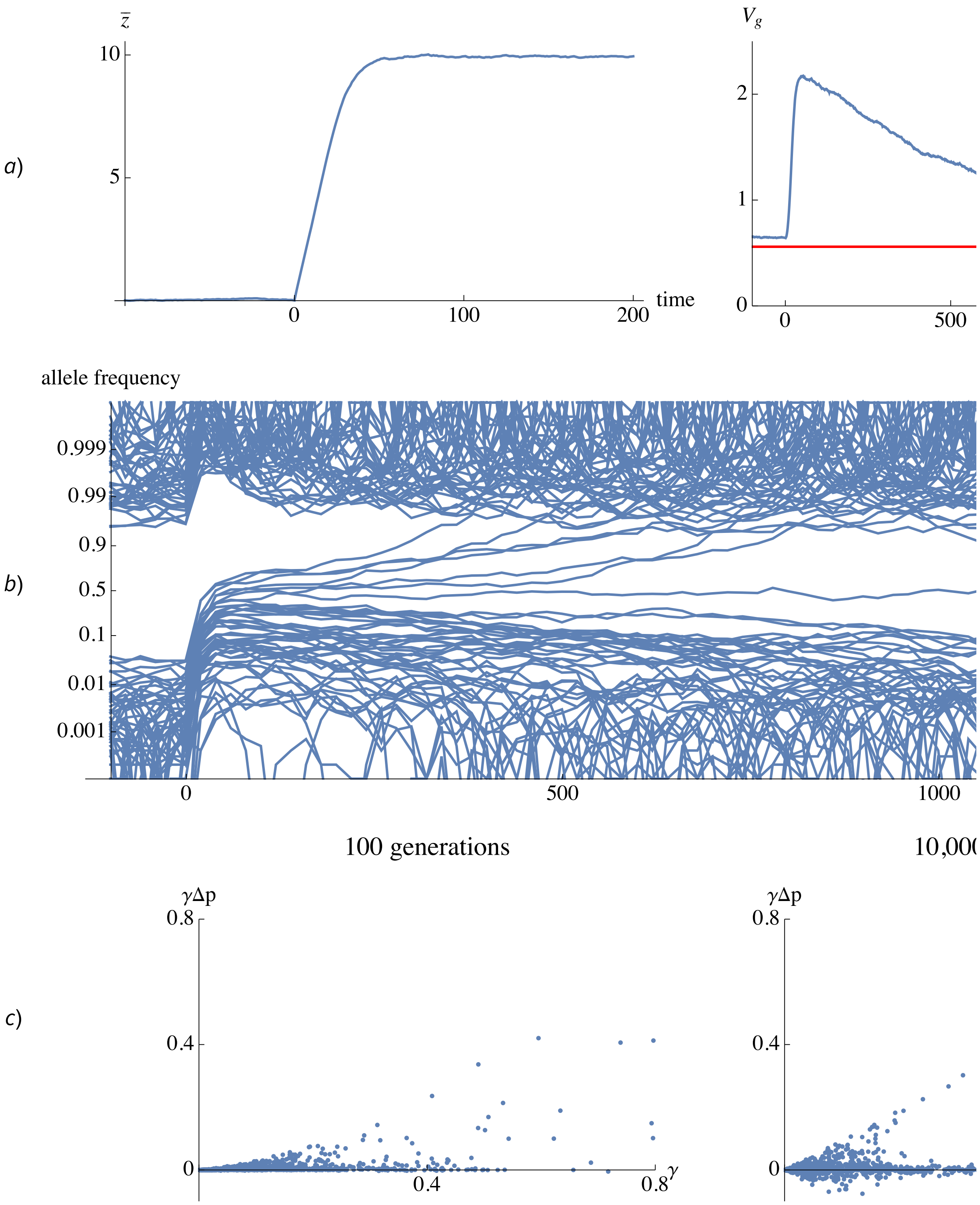

These are the variances at times 0, 100, for all alleles, and then for alleles with γ>0.3

In[∘]:=
```
{a^2.pq0, a^2.pq100, a[[xx]]^2.pq0[[xx]], a[[xx]]^2.pq100[[xx]]}
```

Out[∘]=
```
{0.643834, 2.09163, 0.0803577, 1.35302}
```

These are the changes in mean at 100 and 10^4, for all alleles, and then for alleles with γ>0.3

In[•]:=
```
{a.dp100, a.dp, a⟦xx⟧.dp100⟦xx⟧, a⟦xx⟧.dp⟦xx⟧}
```
Out[•]=
```
{9.61555, 9.68176, 4.11229, 3.8463}
```

These are statistics for substitutions Δp = +1 (γ>0.3); the same, for γ>0.15; the same, for all γ; and for Δp = −1 (all alleles).

In[480]:=
```
xx = Pick[Range[nl], (# > 0.3) & /@ a];
xxs = Pick[Range[nl], (# > 0.15) & /@ a];
subUp = Pick[Range[nl], # > 0.8 & /@ dp];
subDown = Pick[Range[nl], # < -0.8 & /@ dp];
TableForm[Prepend[{Length[#], Total[(a dp)⟦#⟧], Mean[a⟦#⟧], MinMax[a⟦#⟧]} & /@
    {Intersection[subUp, xx], Intersection[subUp, xxs], subUp, subDown},
   {"# subst.", "total Δz̄", "mean Δz̄", "min/max Δz̄"}], TableDepth → 2]
```
Out[481]//TableForm=

# subst.	total Δz̄	mean Δz̄	min/max Δz̄
7	3.81658	0.55361	{0.312748, 0.796693}
16	5.39185	0.351857	{0.153344, 0.796693}
29	6.12985	0.223605	{0.00475523, 0.796693}
3	-0.146233	0.0520901	{0.0303126, 0.0774665}

Definitions

Diffusion

exp4pq

In[•]:=
```
exp4pq::usage = "exp4pq[Nμ,Nsγ^2] gives 𝔼[4p
    q] under the diffusion approximation for haploids";
```

In[•]:=
```
exp4pq[Nμ_, Nsγ2_] :=
  2 Nμ HypergeometriclF1Regularized[2 Nμ + 1, 2 Nμ + 3/2, -Nsγ2 / 4] /
       HypergeometriclF1Regularized[2 Nμ, 2 Nμ + 1/2, -Nsγ2 / 4];
```

In[•]:=
```
{exp4pq[0.1, 1.1], 4 pqbarN[0.1, 1.1, 0]}
```
Out[•]=
```
{0.254171, 0.254171}
```

This checks that $\frac{\partial \langle p\}}{\partial Ns\gamma\Delta} = -2\,\mathrm{var}[p]$

In[•]:=
```
{-2 × 1/4 × (1 - exp4pq[0.1, 1.1]), (pbarN[0.1, 1.1, 0.01] - 0.5) / 0.01}
```
Out[•]=
```
{-0.372915, -0.372906}
```

In[ ]:=
$$\text{FullSimplify}\left[\frac{\int_0^1 (1-\delta^2)^{2\,N\mu}\,\text{Exp}\left[-\text{nsg2v}\left(\frac{1-\delta^2}{4}\right)\right]d\delta}{\int_0^1 (1-\delta^2)^{2\,N\mu-1}\,\text{Exp}\left[-\text{nsg2v}\left(\frac{1-\delta^2}{4}\right)\right]d\delta},\ N\mu>0\right]$$

Out[ ]=
$$\frac{2\,N\mu\ \text{Hypergeometric1F1Regularized}\left[1+2\,N\mu,\ \frac{3}{2}+2\,N\mu,\ -\frac{\text{nsg2v}}{4}\right]}{\text{Hypergeometric1F1Regularized}\left[2\,N\mu,\ \frac{1}{2}+2\,N\mu,\ -\frac{\text{nsg2v}}{4}\right]}$$

intN

In[ ]:=
```
intN::usage =
  "intN[{a,b},Nμ,Nsγ^2,NsγΔ] gives the numerical integral over the
    stationary distribution of p^a(1-p)^b ψ";
```

In[ ]:=
```
intN[{a_, b_}, Nμ_, Nsγ2_, NsΔγ_] := Module[{p},
   NIntegrate[p^(a+2 Nμ-1) (1 - p)^(b+2 Nμ-1) Exp[-Nsγ2 p (1 - p) - NsΔγ (2 p - 1)], {p, 0, 1}]];
```

pqbarN, pbarN

In[ ]:=
```
pqbarN::usage = "pqbarN[{a,b},Nμ,Nsγ^2,NsγΔ] gives 𝔼[pq] numerically";
```

In[ ]:=
```
pqbarN[Nμ_, Nsγ2_, NsΔγ_] := intN[{1, 1}, Nμ, Nsγ2, NsΔγ] / intN[{0, 0}, Nμ, Nsγ2, NsΔγ];
```

In[ ]:=
```
pbarN::usage = "pbarN[{a,b},Nμ,Nsγ^2,NsγΔ] gives 𝔼[p] numerically";
```

In[ ]:=
```
pbarN[Nμ_, Nsγ2_, NsΔγ_] := intN[{1, 0}, Nμ, Nsγ2, NsΔγ] / intN[{0, 0}, Nμ, Nsγ2, NsΔγ];
```

gradP

In[ ]:=
```
gradP::usage =
  "gradP[{γ1,…},Nμ,Ns] gives the gradient of z̄ wrt Δ, for small Δ";
```

In[ ]:=
```
gradP[γ_List, Nμ_, Ns_] := Total[(-2 Ns #^2 (1/4) × (1 - exp4pq[Nμ, Ns #^2])) & /@ γ];
```

pSol

In[ ]:=
```
pSol::usage = "pSol[p0,γ,{Δ0,…},Vs] stoves an interpolation function
    for the deterministic trajectory of allele frequencies";
```

In[•]:=
```
pSol[p0_, γ_, Δ_List, Vs_] := pSol[p0, γ, Δ, Vs];
pSol[p0_, γ_, Δ_List, Vs_, mu_] :=
  pSol[p0, γ, Δ, Vs, mu] = Module[{p, t, del, tm = Length[Δ] - 1},
    del = Interpolation[Transpose[{Range[0, tm], Δ}]];
    p /. NDSolve[
       {∂t p[t] == mu (1 - 2 p[t]) + γ^2/(2 Vs) p[t] (1 - p[t]) × (2 p[t] - 1 - 2/γ del[t]),
        p[0] == p0}, p, {t, 0, tm}][[1]]];
```

tEff

In[•]:=
```
tEff::usage =
  "tEff[p0,γ,{Δ0,…},Vs] stores the cumulative  -t-2/γ ∫_0^t Δⅆt-2/(sγ^2) log[q0/p0],
    which must exceed zero for the allele to increase";
```

In[•]:=
```
tEff[p0_, γ_, Δ_List, Vs_] :=
  tEff[p0, γ, Δ, Vs] = FoldList[Plus, 0, -1 - 2/γ Δ] - (2 Vs)/γ^2 Log[1/p0 - 1];
```

Simulation

initPop

In[•]:=
```
initPop::usage =
  "initPop[nloci,ninds] initialises a population, fixed for one or
    other allele with equal frequency. Returns
    a list nloci with entries 0 or ninds. ";
```

In[•]:=
```
initPop[nl_, ni_] := ni RandomInteger[BernoulliDistribution[1 / 2], {nl}];
```

initP

In[•]:=
```
initP::usage =
  "initP[Nμ,Ns,{γ1,…},ninds] initialises at the diffusion approximation,
    assuming Δ=0, and a stationary distribution.
    initP[Nμ,Ns,{{γ1,1,…},…},ninds] allows for a
    vector of effects, simply by replacing γ by |γ|";
```

In[•]:=
```
initP::badγ = "The list of allelic effects, γ, has the wrong depth,`1`";
```

In[•]:=
```
initP[Nμ_, Ns_, γ_List, ni_Integer] :=
  Module[{xx, pp = Range[1/ni, 1 - 1/ni, 1/ni], pp0 = ni^(-2 Nμ)/(2 Nμ)},
   Switch[Depth[γ], 2 | 3,
    (xx = Join[{pp0}, (pp (1 - pp))^(2 Nμ-1) Exp[- Ns #^2 pp (1 - pp)], {pp0}];
RandomChoice[xx → Range[0, ni]]) & /@ (Norm /@ γ),
    _, Message[initP::badγ, Depth[γ]];
    $Failed]];
```

In order to discretise this, I approximate the ends by the integral from 0 to $p_0 = 1/N$, which is approximately $\frac{p_0{}^{2 N\mu}}{2 N\mu}$

In[•]:=
```
Timing[pp0 = initP[2 ni mu, ni/Vs, a, ni];]
```

Out[•]= {21.1821, Null}

Note that the mean is (by chance) some way from the optimum:

In[•]:=
```
bc = BinCounts[pp0, {0, 1 + 10^4, 10}]; ListLogPlot[0.1 + bc]
{zbar[pp0, a, ni], Vg[pp0, a, ni]}
```

Out[•]=

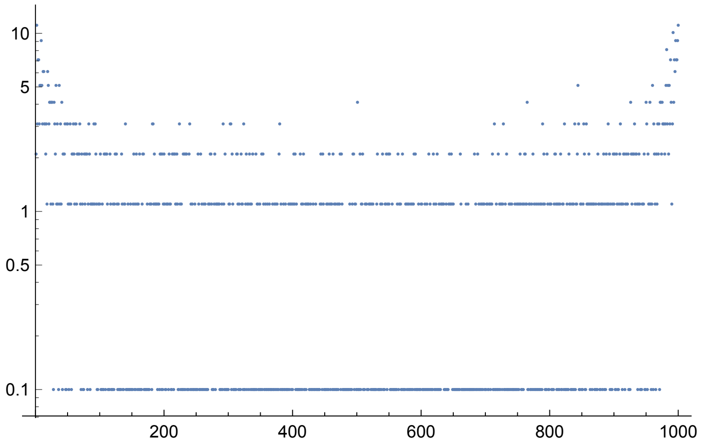

Out[•]= {4.34726, 1.21451}

iterate

In[•]:=
```
iterate::usage = "iterate[{j₁,…},θ,{γ₁,…},Vs,μ,ninds] or
   iterate[{j₁,…},θ,{{γ1,1,…},…},Vs,μ,ninds] iterates through one
   generation of stabilising selection, followed by mutation and drift.
   With ϵ as the last argument, a Poisson/Gaussian approximation to
   the binomial is used. Using Vs=∞ gives a neutral simulation. ";
```

```
In[•]:= iterate[pop_List, θ_, γ_List, Vs_, μ_, ni_Integer] :=
          Module[{zb = zbar[pop, γ, ni]},
           RandomInteger[BinomialDistribution[ni, #]] & /@
            newP[pop, seln[pop, zb - θ, γ, Vs, ni], μ, ni]];
```

```
In[•]:= iterate[pop_List, θ_, γ_List, Vs_, μ_, ni_Integer, ϵ_] :=
          Module[{zb = zbar[pop, γ, ni]},
           randomBinomialApprox[newP[pop, seln[pop, zb - θ, γ, Vs, ni], μ, ni], ni, ϵ]];
```

```
In[•]:= iterate[pop_List, θ_, γ_List, ∞, μ_, ni_Integer] :=
          Module[{zb = zbar[pop, γ, ni]},
           RandomInteger[BinomialDistribution[ni, #]] & /@ newP[pop, 0, μ, ni]];
```

```
In[•]:= iterate[pop_List, θ_, γ_List, ∞, μ_, ni_Integer, ϵ_] :=
          Module[{zb = zbar[pop, γ, ni]},
           randomBinomialApprox[newP[pop, 0, μ, ni], ni, ϵ]];
```

newP

```
In[•]:= newP::usage =
          "newP[{j1,…},{s1,…},ninds] gives the allele frequencies after selection;
            newP[{j1,…},{s1,…},μ,ninds] includes symmetricmutation.
            newP[{j1,…},0,ninds] gives the neutral case.";
```

```
In[•]:= newP[pop_List, 0, ni_Integer] := pop / ni;
        newP[pop_List, 0, μ_, ni_Integer] := μ + (1 - 2 μ) newP[pop, 0, ni];
```

```
In[•]:= newP[pop_List, s_List, ni_Integer] := Module[{es = Exp[-s]},
                    pop
           ─────────────────────── ];
          (1 - es) pop + es ni
        newP[pop_List, s_List, μ_, ni_Integer] := μ + (1 - 2 μ) newP[pop, s, ni];
```

seln

```
In[•]:= seln::usage =
          "seln[{j1,1,…},z-θ,{γ1,…},Vs,ninds] gives the selection coefficients
            for a population, dimension nindsxnloci.
            seln[{j1,1,…},z-θ,{{γ1,1,…},…},Vs,ninds] allows multiple
            traits, with the same stabilising selection Vs on each.";
```

```
In[•]:= seln::badγ = "The list of allelic effects, γ, has the wrong depth,`1`";
```

In[ ]:=
```
seln[pop_List, dz_, γ_List, Vs_, ni_Integer] := Module[{pmq = 2 pop/ni - 1},
  Switch[Depth[γ],
   2, 1/(2 Vs) (-2 γ dz + γ^2 pmq),
   3, 1/(2 Vs) (-2 γ.dz + (Norm /@ γ)^2 pmq),
   _, Message[seln::badγ, Depth[γ]];
   $Failed]];
```

In[ ]:=
```
Histogram[pl[[1]]]
```

Out[ ]=

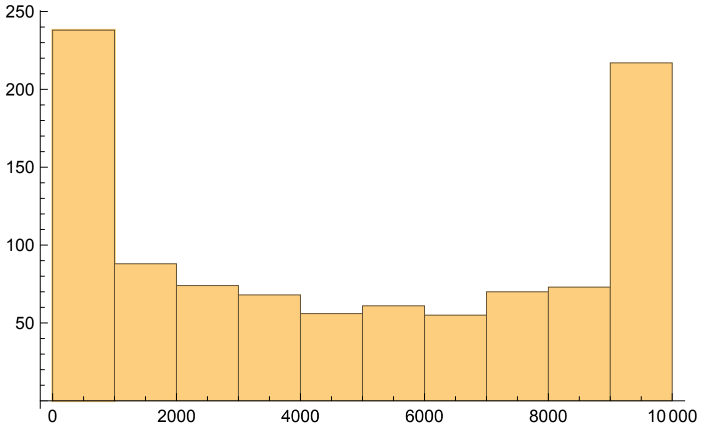

zbar

In[ ]:=
```
zbar::usage =
  "zbar[{j1,1,…},{γ1,…},ninds] lists the trait mean z̄=∑γi (pi-1/2),
    given allelic effects αi, and a metapopulation, defined
    by the number of copies of each allele in each deme.
    zbar[{j1,1,…},{{γ1,1,…},…},ninds] allows multiple traits";
```

In[ ]:=
```
zbar[pop_List, γ_List, ni_Integer] := (pop.γ)/ni - Total[γ]/2;
```

Vg

In[ ]:=
```
Vg::usage =
  "Vg[{j1,…},{γ1,…},ninds] lists the trait variance Vg=∑γi²piqi, given
    allelic effects γi, and a population, defined by the number
    of copies of each allele. Vg[{j1,…},{{γ1,1,…},…},ninds]
    gives the genetic covariance for multiple traits.";
```

In[ ]:=
```
Vg::badγ = "The list of allelic effects, γ, has the wrong depth,`1`";
```

In[]:=
```
Vg[pop_List, γ_List, ni_Integer] := Module[{pq = (pop/ni) (1 - pop/ni)},
   Switch[Depth[γ],
    2, pq.γ^2,
    3, Transpose[γ].(pq γ),
    _, Message[Vg::badγ, Depth[γ]];
    $Failed]];
```

Utilities

randomBinomialApprox

In[]:=
```
randomBinomialApprox::usage =
  "randomBinomialApprox[{{p1,1,…},…},n,ϵ] draws random binomials, using the
    Gaussian approximation for ϵ≤p≤1-ϵ, and Poisson at the edges.";
```

In[]:=
```
randomBinomialApprox[p_List, n_Integer, ϵ_] := Module[{nt},
   nt = Round[
     n p + Sqrt[n p (1 - p)] RandomReal[NormalDistribution[0, 1], Dimensions[p]]];
   MapThread[If[# < ϵ, RandomInteger[PoissonDistribution[n #1]],
      If[# > 1 - ϵ, n - RandomInteger[PoissonDistribution[n (1 - #1)]],
       Min[n, Max[0, #2]]]] &, {p, nt}]];
```

partitionList

In[]:=
```
partitionList::usage = "partitionList[{a1,…},{0,3,…}]
    gathers the list into sets of {0,3,…} elements";
```

In[]:=
```
partitionList[s_List, n : {___Integer}] := Module[{cn = FoldList[Plus, 0, n]},
   MapThread[s[[#1 ;; #2]] &, {Drop[cn + 1, -1], Drop[cn, 1]}]];
```

randomVector

In[]:=
```
randomVector::usage =
  "randomVector[n] makes a random vector, length 1, with n
    dimensions. randomVector[n,k] makes k such vectors.";
```

In[]:=
```
randomVector[n_Integer] := First[randomVector[n, 1]];
randomVector[n_Integer, k_Integer] :=
  Module[{rv = RandomReal[NormalDistribution[0, 1], {k, n}]},
   rv / (Norm /@ rv)];
```
